# A cellular and proteomic approach to assess proteins extracted from cryopreserved human amnion in the cultivation of corneal stromal keratocytes for stromal cell therapy

**DOI:** 10.1186/s40662-019-0155-0

**Published:** 2019-10-12

**Authors:** Beau J. Fenner, Nur Zahirah B. M. Yusoff, Matthias Fuest, Lei Zhou, Francisco Bandeira, Howard Y. Cajucom-Uy, H. K. Tan, Jodhbir S. Mehta, Gary H. F. Yam

**Affiliations:** 1 Tissue Engineering and Stem Cell Group, Singapore Eye Research Institute, 20 College Road, The Academia, Discovery Tower Level 6, Singapore, 169856 Singapore; 2 Singapore National Eye Centre, Singapore, Singapore; 3 Department of Ophthalmology, RWTH Aachen University, Aachen, Germany; 4 Eye-Academic Clinical Programme, Duke-NUS Graduate Medical School, Singapore, Singapore; 5 Proteomics Platform, Singapore Eye Research Institute, Singapore, Singapore; 6 Federal University of São Paulo, Sao Paulo, Brazil; 7 Singapore Eye Bank, Singapore, Singapore; 8 Department of Obstetrics and Gynaecology, Singapore General Hospital, Singapore, Singapore

**Keywords:** Amnion extract, Proteomics, Corneal stromal keratocytes, Marker expression

## Abstract

**Background:**

Human corneal stromal keratocytes propagated in culture media supplemented with human amnion extract (AME) can correct early corneal haze in an animal model. Clinical application of cultivated keratocytes is limited by infectious disease screening before amnion products can be used in humans. It remains unclear if AME from cryopreserved versus fresh human amnion can support human keratocyte propagation, and which components of the extract promote keratocyte growth.

**Methods:**

Three placentas were collected for the preparation of fresh and cryopreserved amnion tissues followed by homogenization and protein extraction. AME protein profiles were studied using isobaric tagging for relative and absolute quantitation (iTRAQ) proteomics. Enriched gene ontology (GO) terms and functional classes were identified. Primary human keratocytes from 4 donor corneas were cultured in media supplemented with fresh AME (F-AME) or cryopreserved AME (C-AME). Cell viability, proliferation and keratocyte marker expression were examined by confocal immunofluorescence and flow cytometry.

**Results:**

AME proteomics revealed 1385 proteins with similar expression levels (between 0.5- and 2-fold) between F- and C-AME, while 286 proteins were reduced (less than 0.5-fold) in C-AME. Enriched GO term and biological pathway analysis showed that those proteins with comparable expression between F-AME and C-AME were involved in cell metabolism, epithelial-mesenchymal transition, focal adhesion, cell-extracellular matrix interaction, cell stress regulation and complement cascades. Human corneal stromal keratocytes cultured with F-AME or C-AME showed similar morphology and viability, while cell proliferation was mildly suppressed with C-AME (*P* > 0.05). Expression of aldehyde dehydrogenase 3A1 (ALDH3A1) and CD34 was similar in both cultures.

**Conclusion:**

AME from cryopreserved amnion had limited influence on keratocyte culture. It is feasible to use protein extract from cryopreserved amnion to propagate human keratocytes for potential translational applications.

## Background

A transparent cornea, which allows uninterrupted passage of light to the retina, is the basis for normal vision. The corneal stroma, which makes up about 90% of the corneal volume, consists of highly organized extracellular matrix (ECM) interspersed with corneal stromal keratocytes (CSKs), contributing to the physical strength and optical properties of the cornea [1]. Corneal opacification, due to scarring and opacities inside the corneal stroma, is a significant cause of global blindness [2, 3]. This reduces and distorts light passage, leading to decreased vision or visual loss. In most situations, surgical removal (corneal transplantation) is commonly used to restore the vision of patients with opacification, such as in keratoconus, post-infection scarring. Recent advances in eye banking and surgical techniques (lamellar keratoplasty), have offered practical advantages over penetrating keratoplasty (such as shorter surgery time, faster recovery and less rejection risk), however, the treatment approach is still limited by the global shortage of donor corneal tissue, long-term graft survival, immune response and the need of surgical expertise [4–6]. Hence, the development of robust strategies, like cell-based therapies, is desirable to restore stromal functions and corneal transparency for patients with corneal opacities [7–11].

Our group has previously reported on the use of intrastromal injection of CSKs to arrest corneal haze development and restore corneal clarity in a rat model of early corneal opacities [12]. Stromal cell-based strategies require ex vivo propagation of CSKs. However, CSKs obtained from donor corneas are challenging to propagate under standard culture conditions [13, 14]. Using serum and growth factor-supplemented media, CSKs rapidly differentiate into stromal fibroblasts (SFs) and lose specific keratocyte features, including the expression of keratan sulphate proteoglycans (lumican, keratocan), which regulates collagen fibril alignment and spacing, and stromal crystallins (transketolase, aldehyde dehydrogenase ALDH1A1 and 3A1) for transparency and refractivity [15, 16]. This irreversible change has created obstacles to the application of cultured stromal cells as a medically useful corneal stromal replacement. SF injection to normal rodent corneas deposited fibrotic ECM proteins to increase light scattering, resulting in haze development [12, 17]. The elevated metalloproteinase levels released by fibroblasts also triggered neovascularization [12]. Hence, the use of correct stromal cell type (i.e., CSKs) is crucial for corneal stromal therapy.

Our group has described the ex vivo propagation of human CSKs using culture medium supplemented with human amnion extract (AME), Rho-associated coiled-coil containing protein kinase (ROCK) inhibitor (Y27632) and insulin-like growth factor 1 (IGF1) (known as ERI supplement) [18]. In the presence of low serum levels, CSKs slowly propagated as “activated keratocytes” without transiting into SFs. After serum withdrawal, they re-expressed keratocyte markers, including lumican, keratocan, ALDH1A1, 3A1, collagen 8A2, CHST6 and B3GNT7. This protocol requires AME as an essential component to suppress transforming growth factor β (TGFβ)-mediated fibroblast transition [18].

Human amnion is known to exert anti-inflammatory, anti-microbial and anti-scarring effects, secrete immunosuppressive factors and promote epithelial wound healing [19–27]. Amnion stroma has been shown to contain growth factors and bioactive substances in regulating TGFβ signalling that support CSK propagation [14, 18, 28, 29]. It remains unknown whether amnion cryo-storage interferes with its ability to promote CSK growth in culture. In this work, we have examined the effects of cryopreservation on AME for human CSK culture and the changes of AME proteomics after cryopreservation.

## Methods

### Human amniotic membrane collection

Human placentas (*n* = 3, foetuses of either gender) were collected after elective caesarean section with written consent from mothers (age < 40 years old), using a protocol approved by our institutional review board (2015/2607, SingHealth, Singapore). After multiple rinses with sterile saline to remove blood traces, the amnion was isolated from the chorion. The amnion portion proximal to the placenta was collected and trimmed into approximately 1 cm^2^ segments. Half of amnion pieces were processed immediately for fresh amnion extract (F-AME), and the remainder were cryopreserved in Dulbecco’s modified Eagle medium (DMEM, Invitrogen, Carlsbad, CA, US) containing 50% glycerol (Sigma-Aldrich, St Louis, MI, US) at − 80 °C for a week and processed for cryopreserved amnion extract (C-AME).

### AME preparation

Amnion pieces were washed with sterile phosphate buffered saline (PBS, 0.1 M, Invitrogen), drip-dried, weighed, and ground under the air phase of liquid nitrogen. The homogenate was agitated at 100 r.p.m. with sterile PBS (5 ml/g tissue) for 48 h at 4 °C. The suspension was passed through a 70 μm filter (Falcon, Corning, NY, US) and centrifuged at 3000 *g* for 20 min. The supernatant was further spun at 48,000 *g* for 20 min at 4 °C. The clear liquid was collected, aliquoted and stored at − 80 °C. An aliquot of each sample was used for protein quantification using Protein DC assay (Bio-Rad, Hercules, CA, US), expression of human tissue inhibitor of metalloproteinase 1 (TIMP1 enzyme-linked immunosorbent assay (ELISA); Invitrogen) and protein mass profiling by sodium dodecylsulfate-polyacrylamide gel electrophoresis (SDS-PAGE). Samples were denatured in buffer containing 50 mM Tris-HCl, 2% SDS, 1% β-mercaptoethanol, 5% glycerol and bromophenol blue (all chemicals procured from Sigma-Aldrich) and resolved using gradient SDS-PAGE (4–20% gradient; Bio-Rad). Gels were stained with Coomassie-blue G-250 and visualized under bright field imaging (GelDoc, Bio-Rad).

### Quantitative AME protein analysis

Protein samples were quantitatively analysed using isobaric tagging for relative and absolute quantitation iTRAQ combined with one-dimensional nano liquid chromatography (LC)-nano-electrospray ionization (ESI)-mass spectrometry (MS)/MS. Analytical runs were performed in triplicates. After denaturation in 50 mM ammonium bicarbonate solution, 2% SDS and *Tris* (2-carboxyethyl) phosphine (TCEP) (reagents in iTRAQ kit, AB Sciex, Framingham, MA, US) for 1 h at 60 °C, protein samples were transferred to a 30 kDa cut-off membrane cartridge (Expedeon, San Diego, CA, US) for concentration using 75% urea solution. They were alkylated with methylmethane thiosulfonate (MMTS; AB Sciex), washed with 75% urea and 50 mM ammonium bicarbonate solution prior to trypsin digestion, which was performed under a substrate:enzyme ratio of 1:25 for overnight. After elution with ammonium bicarbonate and sodium chloride, proteins were labelled with iTRAQ reagents for 3 h, dried and desalted with ultramicrospin columns (Nest Group, Southboro, MA, US).

Labelled proteins were analysed using one-dimensional nano LC-MS/MS (Dionex Ultimate 3000 Nano LC system; ThermoFisher Sci, Sunnyvale, CA, US), coupled with AB Sciex TripleTOF 5600 system (AB Sciex). A Dionex Acclaim PepMap RSLC C18 packed column (ThermoFisher Sci) was connected to a spray tip (New Objective, Woburn, MA, US). They were loaded to a Dionex Acclaim PepMap 100 C18 column (ThermoFisher Sci) and washed with acetonitrile (2/98, vol/vol), acetonitrile (ACN)/water with 0.1% formic acid, followed by a step linear gradient of mobile phase B (2/98 vol/vol of water/ACN with 0.1% formic acid) starting from 7 to 24% for 57 min, to 24–40% for 27 min, to 40–60% for 7 min, and 60–95% for 1 min, at a flow rate of 300 nl/min. The TripleTOF 5600-MS was setup as: ionspray voltage floating = 2400 V, curtain gas = 30, ion source gas 1 = 12, interface heater temperature = 125 °C, declustering potential = 100 V. Data were acquired using information-dependent acquisition (IDA) mode with Analyst TF1.5 (AB Sciex). TOF-MS scan parameters were: 0.25 s accumulation time at the mass range of 350 to ~ 1250 Da followed by product ion scan of 0.05 s accumulation time at the mass range of 100 to 1500 Da. Switching criteria were set to ions greater than m/z = 350 and smaller than m/z = 1250 with charge state of 2 to 5, and an abundance threshold of greater than 120 counts/s. Former target ions were excluded for 12 s and former ions were excluded after one repeat. Maximum number of candidate ions per cycle was 30 spectra. IDA advanced “rolling collision energy (CE)” and “adjust CE when using iTRAQ reagent” were required.

### AME proteomic data analysis

Data were processed and searched against the IPI Human v3.77 database (115,194 proteins) using ProteinPilot 4.1 (AB Sciex). For protein identification, the confidence level was set at 95% and false discovery rate (FDR) less than 1%. Reverse search strategy was used to calculate FDR. For relative quantification, ProteinPilot with the Pro Group algorithm was used to calculate the reporter ions’ peak areas. Automated bias correction was applied to eliminate possible pipetting error during sample preparation.

### Differential protein expression, pathway and statistical analyses

Each sample was analysed in duplicates by iTRAQ, and only data points that were within the 30% coefficient of variance value were used for analysis with outlier exclusion. Expression ratios of protein in C-AME over F-AME were calculated. Cut-off values of ≥2-fold and ≤ 0.5-fold between samples were used. Proteins with changes less than these were considered unaffected. Only proteins with consistent changes in all amnion storage pairs were analysed.

#### Gene ontology (GO) and functional class enrichment analyses

GO terms were determined using GO Resource and PANTHER [30]. Overrepresentation of functional classes was determined with the Database for Annotation, Visualization and Integrated Discovery (DAVID) Bioinformatics Resource v6.8, with a Benjamini statistic cut-off of 0.05 being used to determine statistical significance of overrepresented classes.

### Donor human corneas and primary keratocyte culture

Clinical grade cadaveric corneal tissues (*n* = 4) were procured from Lions Eye Institute for Transplant and Research Inc. (Tampa, FL, US) following institutional review board approval in accordance with approved guidelines. Consent was taken at the time of retrieval by the next of kin, for use in research. Mean donor age was 53.7 ± 7.2 years old and the male-to-female ratio was 1:1. They were preserved in Optisol-GS (Bausch & Lomb Surgical, Irvine, CA, US) and transported at 4 °C to the culture facility. The central button (8 mm diameter) was trephined and treated with dispase II (20 mg/ml; Roche, Basal, Switzerland) followed by gentle scrapping to completely remove corneal epithelium and endothelium. The stromal tissue was digested with collagenase I (1 mg/ml; Worthington, Lakewood, NJ, US) for 6–8 h at 37 °C. Single cells were washed and plated on collagen I-coated culture surface using CSK propagation medium added with ERI supplements and serum (SERI), comprised of DMEM/F12 with L-glutamate (2 mM), HEPES (20 mM), sodium pyruvate (1 mM, Sigma), insulin–transferrin–selenate (1%, Invitrogen), antibiotics-antimycotic (penicillin S, streptomycin sulphate and amphotericin B, Invitrogen), and supplemented with L-ascorbate 2-phosphate (1 mM, Sigma), Y27632 (1 μM; Millipore), insulin-like growth factor 1 (10 ng/ml; Invitrogen) and 0.5% fetal bovine serum (FBS, Gibco) [18]. The media were added with either F-AME or C-AME (5 μg protein/ml). Fresh media was replenished every 3 days. The cultures were sub-passaged when confluence was about 70%. At passage 4, the cultures were switched to serum-free ERI condition for 7 days. During culture, the cells were cultivated continuously with fresh or cryopreserved AME.

### Cell viability - Calcein AM assay

At day 7 of serum-free ERI culture, calcein AM and ethidium homodimer-1 (EthD-1) were added to incubate for 45 min following the Live/Dead Viability/Cytotoxicity kit protocols (Life Technologies). After washes, the samples were mounted in Fluoroshield (Santa Cruz Biotech, Santa Cruz, CA, US) and viewed with a 10x objective under fluorescence microscopy (AxioImager Z1, Carl Zeiss, Oberkochen, Germany). In a minimum of 6 random fields, the number of live (green fluorescence) and dead cells (red fluorescence) was quantified and the percentage of cell viability was calculated and represented as mean ± SD. Experiments were performed in triplicates.

### Cell proliferation - click-iT EdU assay

At day 5 of serum-free ERI culture, EdU (10 μM, Life Technologies) was added and incubated for 48 h. Cells were fixed with 4% paraformaldehyde (Sigma), permeabilized with 0.5% Triton X-100 (Tx, Sigma) and blocked with 3% bovine serum albumin (BSA, Sigma). Click-iT reaction solution (Life Technologies) was added to cells for 30 min. After washes, samples were mounted in DAPI-added Fluoroshield and viewed under fluorescence microscopy with a 10x objective. In a minimum of 6 random fields, the cell proliferation indices were calculated as the percentages of EdU-labelled nuclei and presented as mean ± SD. Experiments were performed in triplicates.

### Flow cytometry

After serum-free ERI culture, CSKs were fixed with 2% paraformaldehyde (Sigma), permeabilized and blocked by 1% Tx, 2% BSA and 2% normal goat serum (NGS, Invitrogen). Cell samples were incubated with rabbit anti-human ALDH3A1 antibody (Proteintech, Rosemont, IL, US), APC-conjugated CD34 antibody (ThermoFisher) or isotype-specific IgG (BD Biosciences, Singapore), followed by FITC–conjugated IgG secondary antibody and propidium iodide. Results were analysed by FACSVerse (BD Biosciences) using a minimum of 10,000 events per experiment. Percentages of positively labelled cells were calculated using FACSuite (BD Biosciences, Singapore).

### Immunofluorescence, ultra-wide field confocal microscopy, z-series reconstruction, cell quantification

Cells on coverslips were fixed with 2% paraformaldehyde, quenched with ice-cold 50 mM ammonium chloride (Sigma-Aldrich) and permeabilized with 0.15% saponin (Sigma-Aldrich). After blocking with 1% BSA and 2% NGS, they were incubated with polyclonal antibodies against ALDH3A1 and CD34 (Millipore), respectively, for 2 h at room temperature. After washes, the labelling signal was revealed by secondary antibody conjugated with AlexaFluor 488 or AlexaFluor 594 (Jackson InnumoResearch Lab, West Grove, PA, US). Samples were mounted in DAPI-added Fluoroshield and viewed under ultra-wide field spinning disk laser confocal microscopy (CSU W1, Nikon) using the “scan large image” mode (NIS Elements, v.4.40). Scan boundaries were marked at 10x magnification with the pixel size set at 0.64 × 0.64 μm and serial z-stacks at 2 μm. Mosaic images were acquired and automatically stitched with XY overlap at 15%. Under a maximum intensity projection function, all z-stacks were merged into a single 2D image. Each culture was performed on 5 coverslips. On each coverslip, at least 5 random fields of 500 × 500 μm were selected for quantifying the immunostained cells and cell percentages against total number of cells (DAPI-labelled) were calculated. Overall percentage was presented as mean ± SD.

### Statistical analysis

Paired Mann-Whitney U (Wilcoxon rank-sum) tests were used to compare cell viability, proliferation rates and percentages of cells expressing keratocyte markers between F-AME and C-AME-supplemented cultures. Results were described as mean ± SD. Statistics were performed using SPSS 20.0 (SPSS, Chicago, IL, US) and Prism 8.0 (GraphPad, San Diego, CA, US). *P* < 0.05 was considered statistically significant.

## Results

### Proteomic profiles of C-AME versus F-AME

**Fig. 1:**
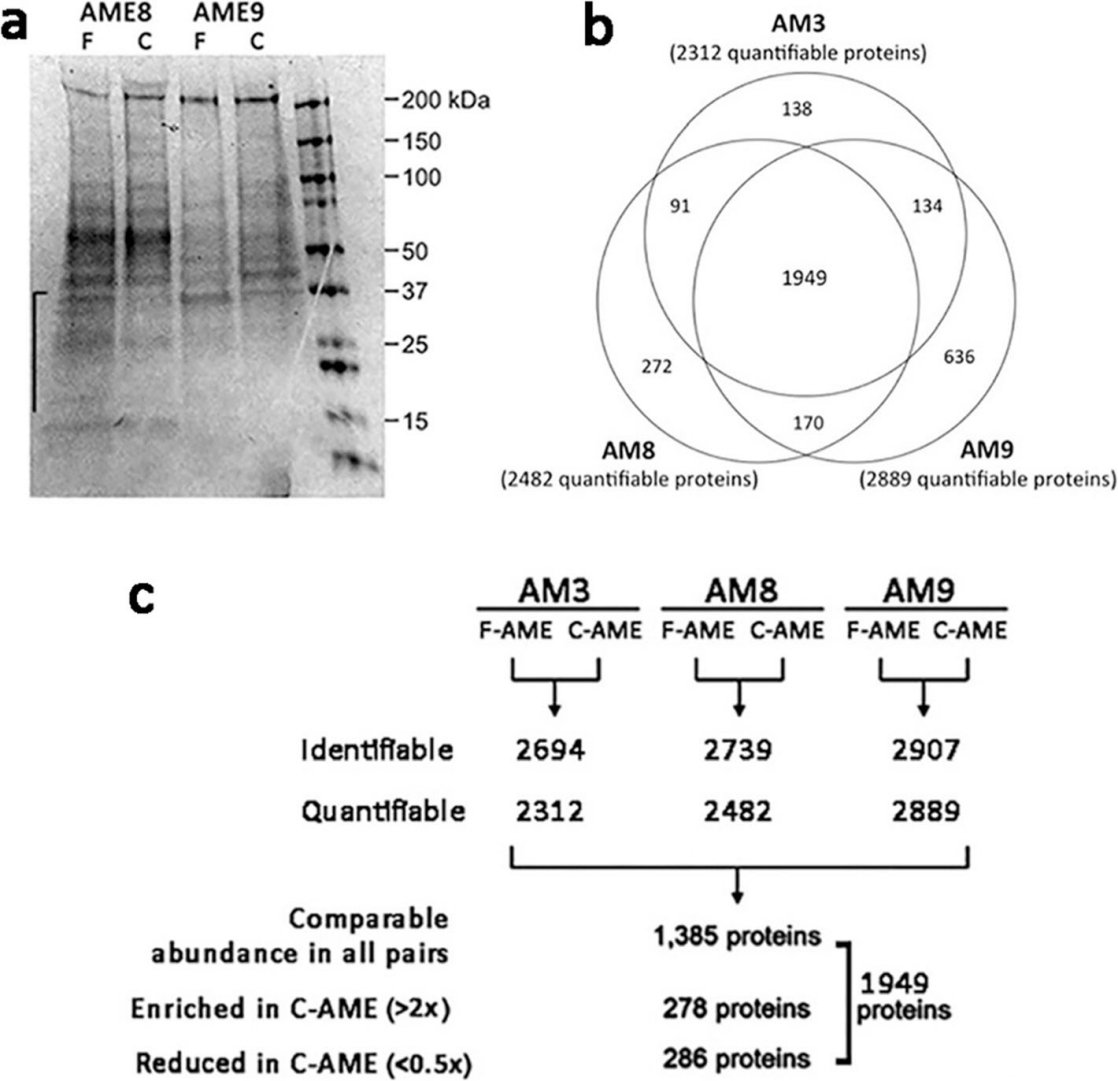
Soluble amnion extract protein profiles. **a** Denaturing gel electrophoresis of amnion extract proteins from fresh and cryopreserved human amnion (F-AME and C-AME). Twenty micrograms of protein prepared from fresh and frozen amnion of two donor tissues, AME8 and AME94 was subjected to 4–20% SDS-PAGE. Major protein bands distribution was revealed after Coomassie brilliant-259 blue staining. Proteins below molecular mass 40 kDa were relatively depleted in C-AME samples. The right-most lane is molecular size ladder. **b** Pie chart representation of quantifiable protein distribution from 3 amnion samples. **c** Experimental approach for iTRAQ proteomic profiling of F-AME and C-AME obtained from 3 different amnion samples (AM3, AM8, AM9). The amount of identifiable and quantifiable proteins is listed. The number of proteins comparatively present (< 2 and > 0.5 fold), enriched (> 2 folds) and reduced (< 0.5 fold) in all amnion samples are indicated

Soluble AME from 3 donor placentas were prepared with fresh amnion tissue or following cryopreservation at − 80 °C for a week. Inspection of gel electrophoresis results indicated that the process of cryopreservation altered the relative abundances of some proteins, most markedly a relative reduction of proteins with molecular mass below 40 kDa in AME from cryopreserved amnion (Fig. 1a). The quality of F-AME prepared from each amnion sample was comparable based on TIMP1 ELISA (AM3: 47 ng/mg protein; AM8: 41 ng/mg protein; AM9; 64 ng/mg protein).

**Fig. 2:**
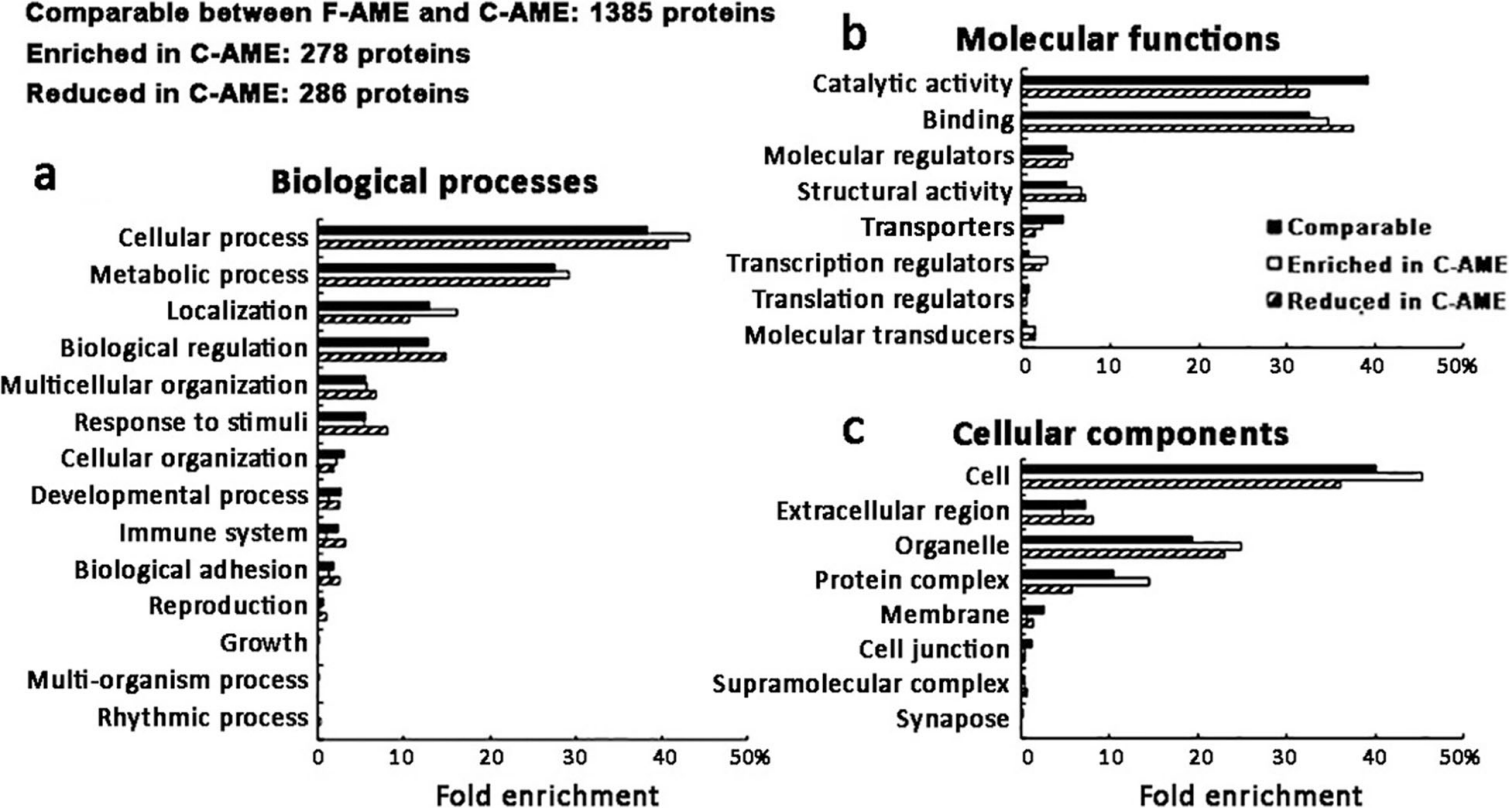
Enriched gene ontology (GO) analyses of extract proteins from fresh and cryopreserved human amnion. The number of proteins comparatively present (< 2 and > 0.5 fold), enriched (> 2 folds) and reduced (< 0.5 fold) in all amnion samples are indicated. Gene ontology distributions for (**a**) biological processes; (**b**) molecular functions; and (**c**) cellular components are illustrated

**Fig. 3:**
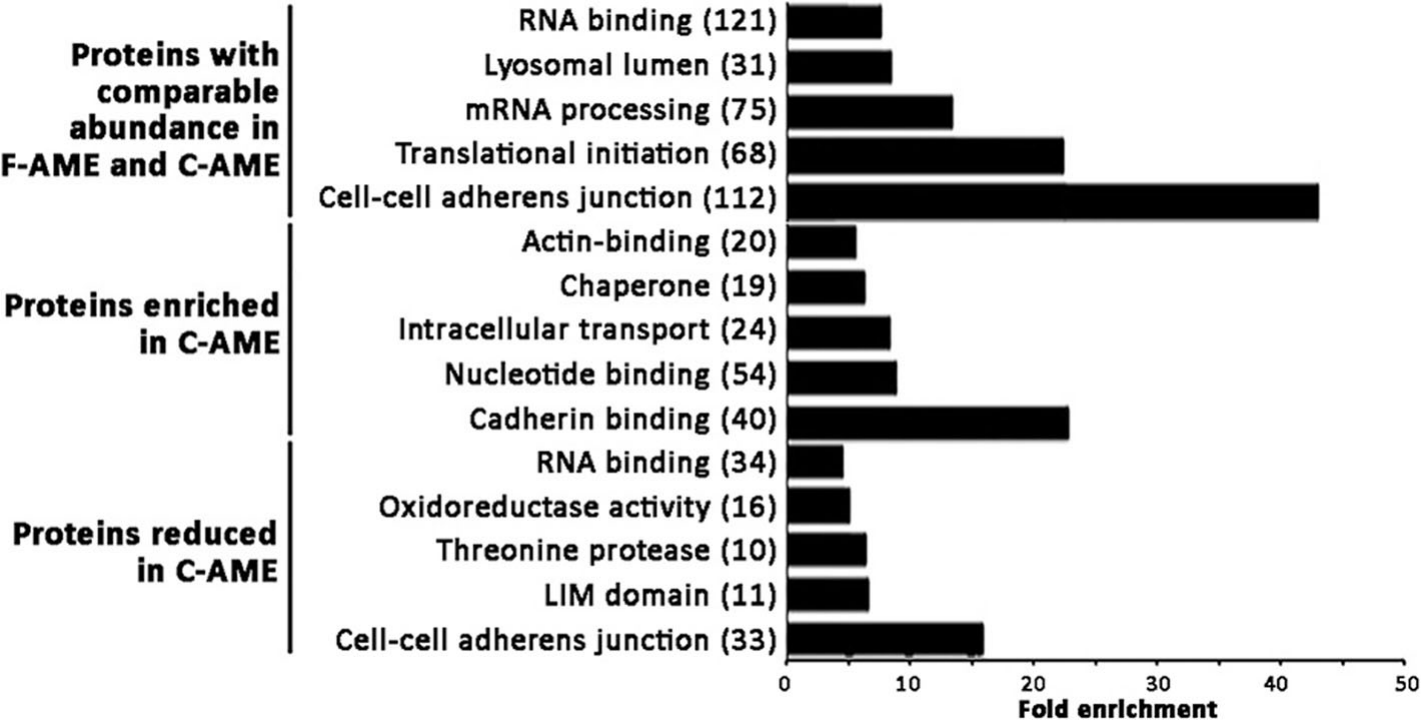
Enrichment analysis of functional protein groups identified by iTRAQ proteomics using amnion extracts derived from fresh or cryopreserved human amnion. Enriched GO analyses were performed and the top 10 enriched functional classes from C-AME proteins that were either of comparable abundance, enriched or reduced relative to F-AME were plotted. The number of discrete proteins for each functional class is shown in parenthesis beside the functional class names. Fold enrichment for each functional class relative to the prevalence of that functional class in the total human proteome is listed on X-axis

Proteomic profiling of F-AME and C-AME yielded an average of 2194 identifiable proteins and 1812 quantifiable proteins in AM3 samples; 2739 identifiable proteins and 1482 quantifiable proteins in AM8 samples; and 2907 identifiable proteins and 2889 quantifiable proteins in AM9 samples (Fig. 1b-c). A comparison of quantifiable proteins was performed by identifying proteins that were enriched or reduced between C-AME and F-AME, yielding 278 discrete enriched proteins (> 2 folds) and 286 reduced proteins (< 0.5 fold) in C-AME. The remaining 1385 proteins were considered unchanged (between 0.5- and 2-fold) between F- and C-AME (Fig. 1c). GO analysis revealed a similar distribution of ontology classes among AME proteins in the comparable, enriched and reduced populations (Fig. 2a-c). Proteins with comparable expression in both F-AME and C-AME were predominantly associated with cellular and metabolic processes in the category of biological processes; catalytic activity and binding in molecular functions; and organelles and protein containing complex in cellular components. Similar trends were seen for enriched and reduced proteins in C-AME compared to F-AME. Some overlaps were found in the over-represented functional classes in C-AME versus F-AME (Fig. 3). Among proteins with unchanged expression levels, they were involved in cell-cell adhesion, translational initiation, and protein and RNA metabolism. Cell-cell adhesion proteins were similarly over-represented among the enriched and reduced proteins in C-AME proteomes.

### AME proteins preserved after AM cryopreservation

Supplementation of culture media with F-AME or C-AME had no significant effect on human CSK viability, proliferation and marker expression. This indicated that proteins with comparable expression levels in F-AME and C-AME (*n* = 1385 proteins) would be important to CSK growth in culture (Supplemental information). Using DAVID functional annotation, the enriched GO terms predicted for these proteins were significantly associated to Fc-epsilon receptor signalling pathway (*P* < 0.001, Benjamini test; enrichment score EnS:6.55), NIK/NF-kappaB signalling (*P* < 0.001; EnS:6.55), regulation of mRNA stability (*P* < 0.001; EnS:6.55), protein polyubiquitination (*P* < 0.001; EnS:3.87), and cell-cell adhesion (*P* = 0.02; EnS:3.63) (Table 1). The significant Kyoto encyclopedia of genes and genomes (KEGG) pathways were proteasome (*P* < 0.01; EnS:6.55), complement and coagulation cascades (*P* = 0.043; EnS:3.43).

**Table 1:** Enriched Gene Ontology terms and KEGG pathways identified for proteins similarly expressed in F-AME and C-AME (between 0.5- and 2-fold) of 3 donor amnion samples

	Enrichment scores	Biological pathways	Proteins (UniProt Accession no.)	*p*
(A) Enriched GO terms (DAVID)				
1	6.55	GO:0038095~Fc-epsilon receptor signalling pathway	P20618, P25789, P25786, P28070, P01700, O14818, P63208, P60900, P49721, P49720, P63098, P0DOY3	2.8E-6*
2	6.55	GO:0038061~NIK/NF-kappaB signalling	P20618, P25789, P25786, P28070, O14818, P63208, P60900, P49721, P49720	2.3E-6*
3	6.55	GO:0043488~regulation of mRNA stability	P20618, P27695, P25789, P25786, P28070, O14818, Q92945, P60900, P49721, P49720	1.9E-6*
4	3.87	GO:0000209~protein polyubiquitination	P20618, P25789, P25786, P28070, O14818, P63208, P60900, P49721, P49720	4.6E-04*
5	3.63	GO:0098609~cell-cell adhesion	P30041, O60437, P62820, P46109, Q92817, Q15907, P43487	0.02*
6	3.12	GO:0000398~mRNA splicing, via spliceosome	P52597, Q08170, P22626, P14866, Q16629, Q86U42	0.19
(B) KEGG pathways (DAVID)				
1	6.55	hsa03050: Proteasome	P20618, P25789, P25786, P28070, O14818, P60900, P49721, P49720	1E-5*
2	3.43	hsa04610: Complement and coagulation cascades	P01008, P02746, P01009, P00488	0.043*
3	3.426	hsa03010: Ribosome	P42766, P46779, P39019, Q969Q0, P62979, P62899, P62917, P62266, Q02878, P42677	0.082
4	3.113	hsa03040: Spliceosome	P26368, O14776, P51991, O75533, Q13435, P62312, O43143, P09012, P08621, Q6P2Q9	0.072

*GO* = gene ontology, *KEGG* = kyoto encyclopedia of genes and genomes, *F-AME* = fresh human amnion extract, *C-AME* = cryopreserved human amnion extract, *DAVID* = database for annotation, visualization and integrated discovery

The biological events were ranked using enrichment scores. *Adjusted Benjamini *p* < 0.05 represents statistical significance

We selected the top 40 abundant proteins expressed in both F-AME and C-AME and examined their predicted roles in CSK cultivation (Table 2). Identification of specific functional classes was performed using the Wilcoxon pathway enrichment analysis component. Identified proteins that were involved in epithelial-mesenchymal transition, focal adhesion, cell-ECM interaction and receptor tyrosine kinase-signalling included serum albumin, filamin B, vimentin, tenascin, moesin, collagen 2A1, 1A1, 3A1, 6A3 and 14A1. Cell metabolism and stress response proteins included serum albumin, filamin B, spectrin α, serotransferrin, prelamin A/C, pyruvate kinase, protein disulfide-isomerase A3, heat shock 71 kDa protein, ezrin, glucose regulated 78 kDa protein, neural α-glucosidase AB, moesin and hydropyrimidinase-related protein 2.

**Table 2:** Top 40 proteins with comparable expression (< 2 and > 0.5 fold) between C-AME and F-AME in all amnion samples

No.	UniProt Accession No.	Protein	Protein symbol	Mean Abundance*	Mean fold changes**
1	P02768	Serum albumin	ALB	457	0.89
2	Q09666	Neuroblast differentiation AHNAK	AHNAK	435	0.89
3	O75369	Filamin-B	FLNB	408	1.03
4	Q99715	Collagen 12A1	COL12A1	407	1.09
5	Q8IVF2	AHNAK Nucleoprotein 2	AHNAK2	242	0.82
6	P12111	Collagen 6A3	COL6A3	207	1.5
7	Q13813	Spectrin α chain	SPTAN1	205	1.64
8	Q15149	Plectin	PLEC	178	0.86
9	P02452	Collagen 1A1	COL1A1	173	0.63
10	P01023	α2-macroglobulin	A2M	172	0.8
11	P68871	Haemoglobin subunit β	HBB	169	0.59
12	P08670	Vimentin	VIM	158	1.99
13	P02787	Serotransferrin	TF	157	0.88
14	P02545	Prelamin-A/C	LMNA	146	1.37
15	A8K2U0	α2-macroglobulin-like protein 1	A2ML1	145	0.75
16	P01024	Complement C3	C3	142	1.08
17	P07355	Annexin A2	ANXA2	124	0.72
18	P06396	Gelsolin	GSN	122	0.91
19	P01861	Immunoglobulin heavy constant γ4	IGHG4	120	0.6
20	P14618	Pyruvate kinase	PKM	113	0.99
21	P01859	Immunoglobulin heavy constant γ2	IGHG2	109	0.56
22	P24821	Tenascin	TNC	108	0.62
23	P02461	Collagen 3A1	COL3A1	108	0.99
24	P30101	Protein disulfide-isomerase A3	PDIA3	98	0.56
25	P11142	Heat shock cognate 71 kDa protein	HSPA8	94	0.89
26	P15311	Ezrin	EZR	94	1.9
27	P07585	Decorin	DCN	92	0.87
28	P11021	78 kDa glucose-regulated protein	HSPA5	92	1.08
29	P63104	14–3-3 protein zeta/delta	YWHAZ	88	0.6
30	P0C0L5	Complement C4-B	C4B	83	0.88
31	P0C0L4	Complement C4-A	C4A	80	0.94
32	P98160	Basement membrane-specific heparan sulphate proteoglycan core protein	HSPG2	80	1.09
33	P05787	Keratin 8	KRT8	77	1.76
34	P08603	Complement factor H	CFH	73	0.64
35	Q14697	Neutral α-glucosidase AB	GANAB	72	1.55
36	P26038	Moesin	MSN	71	0.74
37	Q16555	Dihydropyrimidinase-related protein 2	DPYSL2	71	1.41
38	Q05707	Collagen 14a1	COL14A1	69	1.99
39	P13667	Protein disulfide-isomerase A4	PDIA4	68	0.94
40	P02788	Lactotransferrin	LTF	68	0.72

*F-AME* = fresh human amnion extract, *C-AME* = cryopreserved human amnion extract

*The protein list was ranked by mean abundance. **Mean fold changes from all 3 amnion samples

### Downregulated AME proteins after AM cryopreservation

We identified that 286 proteins were less expressed (< 0.5-fold) in C-AME, when compared to F-AME. We listed the top 40 downregulated proteins in Table 3, and they included mimecan, annexin A2, A4, cathepsin B, α-enolase, heterogeneous nuclear ribonucleoprotein A3, S100A10, triosephosphate isomerase, heat shock 70 kDa protein 1B. Using DAVID functional annotation, the enriched GO terms that significantly linked to the downregulated proteins were cell-cell adhesion (*P* < 0.001; EnS 15.19), mRNA stability (*P* < 0.001; EnS 6.45), proteolysis (*P* < 0.001; EnS 6.45) and glycolytic process (*P* = 0.026; EnS 4.23) (Table 4). Two significant KEGG pathways were predicted, which were proteasome (*P* < 0.01; EnS 6.45) and biosynthesis of antibiotics (*P* = 0.014; EnS 4.23).

**Table 3:** Top 40 proteins with reduced expression (< 0.5 fold) in C-AME, compared to F-AME

No.	UniProt Accession No.	Protein	Protein symbol	Mean fold changes**
1	Q8WUF5	RelA-associated inhibitor	PPP1R13L	0.010
2	P58215	Lysyl oxidase homolog 3	LOXL3	0.040
3	P20774	Mimecan	OGN	0.052
4	P07355	Annexin A2	ANXA2	0.057
5	O95171	Sciellin	SCEL	0.069
6	P51991	Heterogeneous nuclear ribonucleoprotein A3	HNRNPA3	0.081
7	P07203	Glutathione peroxidase 1	GPX1	0.092
8	P09429	High mobility group protein B1	HMGB1	0.098
9	O76041	Nebulette	NEBL	0.100
10	P09525	Annexin A4	ANXA4	0.111
11	P63244	Receptor of activated protein C kinase 1	RACK1	0.112
12	P60174	Triosephosphate isomerase	TPI1	0.113
13	Q9UBC9	Small proline-rich protein 3	SPRR3	0.114
14	P23497	Nuclear autoantigen Sp-100	SP100	0.128
15	O15231	Zinc finger protein 185	ZNF185	0.129
16	P0DMV9	Heat shock 70 kDa protein 1B	HSPA1B	0.130
17	Q92541	RTF1 Homolog, Paf1/RNA Polymerase II Complex Component	RTF1	0.131
18	Q8NC51	Plasminogen activator inhibitor 1 RNA-binding protein	SERBP1	0.134
19	P00338	L-lactate dehydrogenase A	LDHA	0.134
20	O43768	Alpha-endosulfine	ENSA	0.135
21	Q9GZT8	NGG1 Interacting Factor 3 Like 1	NIF3L1	0.136
22	P07858	Cathepsin B	CTSB	0.137
23	P20810	Calpastatin	CAST	0.138
24	P00558	Phosphoglycerate kinase 1	PGK1	0.139
25	P56211	cAMP-regulated phosphoprotein 19	ARPP19	0.140
26	Q9P258	Regulator of Chromosome Condensation 2	RCC2	0.147
27	P50238	Cysteine-rich protein 1	CRIP1	0.148
28	P60903	Protein S100-A10	S100A10	0.149
29	P62937	Peptidyl-prolyl cis-trans isomerase A	PPIA	0.150
30	P04075	Fructose-bisphosphate aldolase A	ALDOA	0.151
31	Q01469	Fatty acid-binding protein	FABP5	0.156
32	P31939	Bifunctional purine biosynthesis protein PURH	ATIC	0.158
33	P12277	Creatine kinase B-type	CKB	0.158
34	P00441	Superoxide dismutase [Cu-Zn]	SOD1	0.161
35	Q8WWI1	LIM domain only protein 7	LMO7	0.163
36	P06733	Alpha-enolase	ENO1	0.164
37	O75083	WD repeat-containing protein 1	WDR1	0.166
38	P10915	Hyaluronan and proteoglycan link protein 1	HAPLN1	0.167
39	O43399	Tumour Protein D52 Like 2	TPD52L2	0.168
40	P09211	Glutathione S-transferase P	GSTP1	0.169

*F-AME* = fresh human amnion extract, *C-AME* = cryopreserved human amnion extract

*The protein list was ranked by mean fold changes from all 3 amnion samples

**Table 4:** Enriched Gene Ontology terms and KEGG pathway identified for proteins with reduced expression in C-AME (< 0.5-fold) compared to F-AME

	Enrichment scores	Biological pathways	Proteins (UniProt Accession no.)	*p*
(A) Enriched GO terms (DAVID)				
1	15.19	GO:0098609~cell-cell adhesion	P07355, P62820, Q8WUF5, P61026, P31947, P06733, P04075, Q9H444, P43487, Q9C0C2, P30041, Q15056, P46109, P31949, Q15907, Q99497, Q9UJU6, O60437, P00338, Q92817, P31939, Q16643, P20810, Q9NYL9, P0DMV9, Q16658, P63244, Q14847, Q8NC51, Q14247	1.3E-12*
2	6.45	GO:0043488~regulation of mRNA stability	P27695, P25788, P25789, P25786, P0DMV9, P20618, P28070, O14818, Q92945, P60900, P49721, P04792, P49720, Q8NC51, P28066	2E-6*
3	6.45	GO:0051603~proteolysis involved in cellular protein catabolic process	P20618, P25788, P25789, P25786, P28070, O14818, P07858, P60900, P49721, P49720	1E-4*
3	4.23	GO:0006096~glycolytic process	P60174, P04406, P00558, P04075, P00338, P06733, P06744	0.026*
(B) KEGG pathways (DAVID)				
1	6.45	hsa03050:Proteasome	P20618, P25788, P25789, P25786, P28070, O14818, P60900, P49721, P49720, P28066	3.6E-4*
2	4.23	hsa01130:Biosynthesis of antibiotics	P40925, P60174, P04406, P37837, P06733, P00338, P04075, Q03154, P06744, P31939, P11766, P54687, P29401, P14324, P00558, P14550, P21399	0.014*

*GO* = gene ontology, *KEGG* = kyoto encyclopedia of genes and genomes, *F-AME* = fresh human amnion extract, *C-AME* = cryopreserved human amnion extract, *DAVID* = database for annotation, visualization and integrated discovery

The biological events were ranked using enrichment scores. *Adjusted Benjamini *p* < 0.05 represents statistical significance

### Keratocyte propagation in culture media supplemented with F-AME or C-AME

**Fig. 4:**
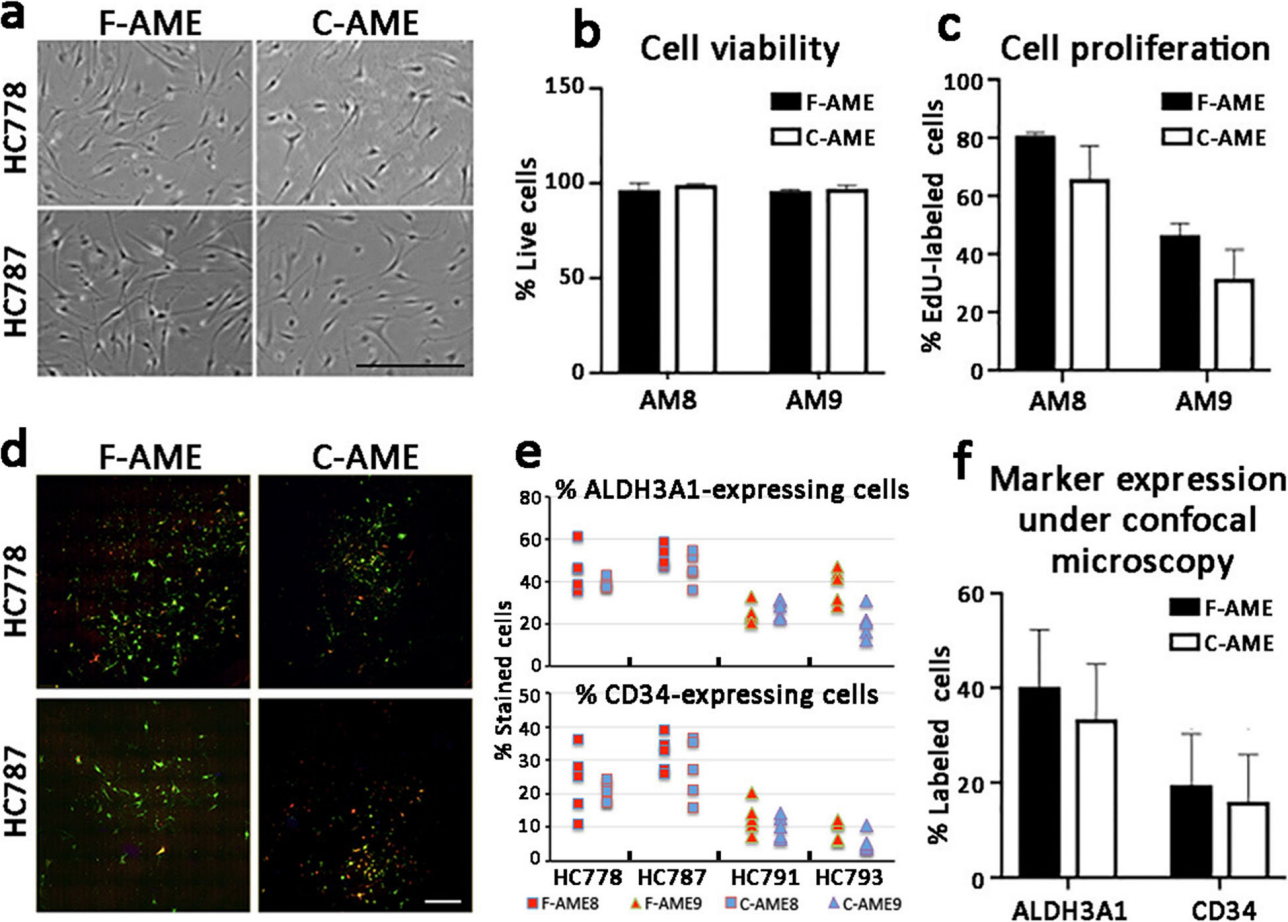
Primary human keratocyte cultures in ERI medium supplemented with F-AME or C-AME. **a** Phase contrast micrographs showing CSK morphology after 5 passages in culture. Primary CSKs were prepared using donor corneal stromal tissue, HC778 and HC787. **b** Cell viability by calcein AM staining and cell quantification. Data are presented as mean and standard deviation for 4 primary CSK cultures. **c** Cell proliferation by EdU incorporation assay and cell quantification (*n* = 4 primary CSK cultures). **d** Wide-field spinning disk confocal laser microscopy for CSKs immunostained for ALDH3A1 (green fluorescence) and CD34 (red fluorescence). **e** Cell quantitation for the percentage of CSKs expressing ALDH3A1 and CD34 by confocal immunofluorescence in 4 primary CSK cultures. **f** Mean percentages of CSKs expressing ALDH3A1 and CD34 by immunostaining. Error bars: standard deviation. Scale bars: 300 μm (**a**), 500 μm (**d**)

**Fig. 5:**
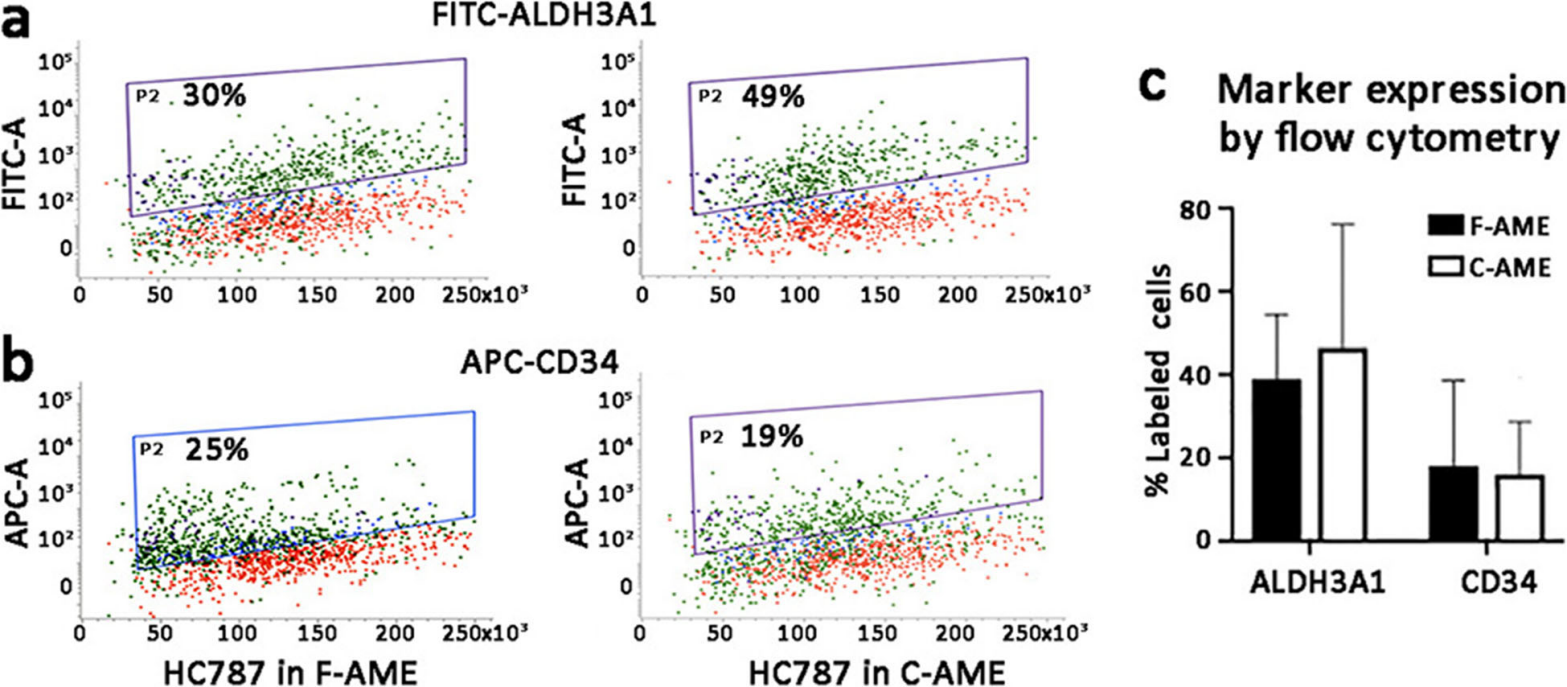
Flow cytometry analysis of CSK marker expression in primary cultures with F-AME or C-AME. Scatter plots of (**a**) ALDH3A1 (FITC-stained) and (**b**) CD34 (APC-conjugated) expression in primary CSK culture with F-AME versus C-AME. **c** Mean percentages of CSKs (*n* = 4 cultures) expressing ALDH3A1 and CD34 by flow cytometry. Error bars: standard deviation

Primary human CSK cultures utilized previously established protocol (see Methods). The cultured cells assumed the characteristic dendritic morphology with cell processes extending to connect with neighbouring cells forming a cellular network (Fig. 4a). Similar cell morphology was observed following culture in media supplemented with either F-AME or C-AME, with good reproducibility among cells harvested from different donor corneas (*n* = 4) and AME pairs (*n* = 2) (Fig. 4a). Cell viability was maintained > 95% at the 5th passage under culture in both F-AME and C-AME-supplemented media (Fig. 4b), with no significant difference between extracts from different donor amnion. The rate of cell proliferation, revealed by EdU incorporation, varied from 20 to 80% depending on primary CSKs from different donors. There was a trend towards lower proliferation in cultures supplemented with C-AME, although this was not significantly different than that seen in F-AME supplemented media (Fig. 4c). Expression of keratocyte markers was assessed by immunofluorescence followed by confocal laser microscopy and flow cytometry, respectively. Cells expressing ALDH3A1 and CD34 were observed in all primary cultures under wide-field confocal imaging (Fig. 4d). Cell quantification showed no significant difference in the mean percentages of ALDH3A1-expressing cells (F-AME culture: 40 ± 12%; C-AME culture: 33 ± 14%) and CD34-expressing cells (F-AME culture: 20 ± 10%; C-AME culture: 17 ± 11%) (Fig. 4e-f). Similarly, no significant difference was observed for percentages of marker-expressing cells after cultivation with F-AME or C-AME under flow cytometry (ALDH3A1-positive cells was 39 ± 14% in F-AME and 44 ± 28% in C-AME culture; whereas CD34-positive cells was 19 ± 21% in F-AME and 17 ± 14% in C-AME culture) (Fig. 5a-c).

## Discussion

In this study, we have validated the ex vivo propagation of human CSKs from donor corneas using an ERI protocol. Culture media supplemented with AME from fresh or cryopreserved amnion did not alter cell morphology, viability and marker expression, although the cell proliferation rate was slightly reduced in cultures with C-AME. Hence, it is feasible to use soluble protein extracts from cryopreserved AM to propagate human CSKs for potential translational applications. We also characterized the proteomes of fresh and cryopreserved amnion tissues by iTRAQ approach and showed that 1385 proteins expressed at comparable levels in both F-AME and C-AME and involved in various cellular pathways, including cell metabolism, epithelial-mesenchymal transition, focal adhesion, cell-ECM interaction, and cell stress regulation (proteasome activity, protein polyubiquitination), and complement cascades.

Development of tissue engineered human corneas remains one of the most important goals in ophthalmology today [6, 31]. A major hurdle to the development of clinically relevant tissue constructs or cell-based therapy is the lack of a widely acceptable source of corneal stromal keratocytes (CSKs), which are crucial for maintaining corneal transparency, refractivity and mechanical strength [32, 33]. Ex vivo culture of human CSKs is now possible with the use of AME and supplements [12, 18], but the safety concerns regarding use of human amnion, such as disease transmission and screening limitations, make this approach potentially difficult. Currently, the complete maternal screening for infectious diseases (e.g., HIV, HBV) takes 6 months post-partum [34, 35]. This necessitates cryopreserving the amnion, typically at − 80 °C, for a period of time before clinical and laboratory uses. In our current work, we have demonstrated that primary human CSKs can be propagated in culture media supplemented with extract from cryopreserved amnion (C-AME). This may overcome the potential limitation of using extracts from freshly harvested amnion (F-AME),

Keratocyte cultures supplemented with C-AME were comparable to those with F-AME, in terms of cell morphology and viability, although we noted a trend towards slower proliferation when using C-AME. Current literature has shown variable outcomes regarding the impact of cryopreservation on the growth-promoting properties of amnion extracts, depending on the biological model being used. Cooke and colleagues reported that cryopreserved amnion extracts retained anti-inflammatory and anti-apoptotic properties in culturing macrophages, while dehydrated amnion lacked these activities [36]. In a mouse model of wound healing, cryopreserved and lyophilized amnion showed comparable pro- healing activity [37]. Vacuum-dried amnion was found superior to cryopreserved amnion for the expansion of corneal epithelial cells, with increased proliferation and reduced apoptosis [38]. In our work, there was a trend towards slower CSK proliferation in cultures with C-AME-supplemented media compared to that of F-AME, though the difference was not statistically significant.

Only a few studies have employed proteomic approaches to examine the protein composition of human amnion. In 2006, Park et al. reported 92 soluble and 19 membrane proteins from amnion using two-dimensional gel electrophoresis and mass spectrometry [39]. Using similar proteomic techniques to assess the protein profile of cryopreserved amnion, Hopkinson and co-workers detected 48 proteins in both the tissue and washing supernatant. They suggested some soluble proteins were released due to the preservation and tissue handling steps, resulting in inter-membrane variation, that could compromise the desired therapeutic effect after amnion transplantation [40]. In this work, we also identified 39 of these 48 proteins, of which 26 were also reduced following cryopreservation (see Supplemental Information). Enriched GO term analysis has indicated that the overall distribution of biological processes, molecular functions and cellular components for C-AME proteins were largely similar to those predicted for F-AME, including cell-cell adhesion and cell metabolism (RNA binding, actin-binding, protein biosynthesis). This indicates that there were no dramatic changes to the overall distribution of extract proteins. For the downregulated proteins in C-AME, they were predicted to associate with several enriched GO terms, which were cell adhesion, mRNA stability and glycolytic process. This might not exert any significant effects on cell metabolism, growth and survival. The deregulated cellular functions might be compensated by proteins that remained in C-AME. Those enriched in C-AME were involved in intracellular and vesicle-mediated transport as well as cell stress response.

Fibroblast transition and growth is detrimental to CSK culture. They behave differently to bona fide CSKs (including morphologic changes, loss of CSK-specific keratan sulphate-proteoglycans and crystallins), and their presence typically results in overgrowth with fibroblasts dominating the cell culture. The use of amnion extract in CSK culture has been associated with the suppression of TGFβ/Smad signalling, which downregulates α-smooth muscle actin (αSMA) and fibronectin expression and prevents fibroblast transformation [18, 28, 41]. Likewise, keratocan expression was preserved when mouse keratocytes were cultured inside the amnion stroma [42]. In this study, proteins present in C-AME also regulated the epithelial-mesenchymal transition, validating the effect on TGFβ signalling and fibroblast development. Other pathways included focal adhesion, cell-ECM interaction and receptor tyrosine kinase signalling, as well as cell stress regulation.

Despite the substantial amnion proteome identified here, the specific proteins responsible for promoting CSK growth in culture remain unknown. We identified approximately 300 secreted proteins in the amnion proteome using human secretome database analysis [43], together with previously described keratocyte growth promoters, including lumican, IGF2 and the binding proteins IGFALS and IGFBP5, being among the secreted proteins. Future work will include the identification of secreted proteins that play a role in the keratocyte growth-promoting properties of amnion extracts.

Use of AME is not limited to keratocyte culture. In a rabbit corneal damage model, topical AME has provided similar effects as that of amnion grafting [44]. It shows greater healing effect than autologous serum in treating corneal alkali injury [45]. It also dramatically reduced corneal inflammation and angiogenesis in an experimental herpes simplex keratitis model by modulating the level of proinflammatory cytokines [46]. Hence, the characterization of bioactive components present in AME could contribute to identify potentially useful AME with optimal therapeutic effects for clinical application.

Potential limitations of this work include variable protein composition of amnion extracts obtained from different donor placentas. The variable proliferation of CSKs with different amnion extracts suggests differences in amnion harvesting, storage, and potentially the physiological state of the donor mother, which may all play a role in determining the efficacy of amnion extracts for supporting CSK growth. Given the biological complexity of the amnion extract, it is also difficult to draw conclusions about the relevance of our proteomic screens, and substantial work remains to identify the growth factors or other bioactive molecules required to propagate CSKs in vitro. Another potential limitation is the use of glycerol as a cryopreservative for amnion prior to extract preparation. Previous work had indicated that even brief periods (weeks) of amnion storage in glycerol-added preservation medium resulted in a rapid loss of cell viability (< 30% viability) [47]. The release of proteins from dead cells in the protein extracts may have contributed to the diminished efficacy for CSK propagation compared to fresh amnion extracts. Alternative cryopreservation in the presence of dimethylsulfoxide (DMSO), a common cryoprotectant to prevent intracellular ice formation, or under freeze-drying with the removal of ice through sublimation, can maintain cell viability [48, 49]. However, our earlier report has revealed a significant loss of growth factors and ECM proteins (such as laminin and fibronectin) in freeze-dried amnion, which might produce the protein extract with reduced potential in supporting cell growth [50]. Additional experiments testing the effect of DMSO or freeze-drying would be useful to determine how the storage protocol influences the utility of amnion extracts for CSK culture.

## Conclusion

In conclusion, we have demonstrated that protein extract from cryopreserved amnion was able to support the survival and proliferation of primary human CSKs. This opens new research avenues for the timely clinical application of cultured keratocytes for treating corneal stromal diseases.

## Data Availability

All data are included in the text and supplemental information.

## Abbreviations

ACN: Acetonitrile
ALDH: Aldehyde dehydrogenase
AME: Amnion extract
BSA: Bovine serum albumin
C-AME: Cryopreserved amnion extract
CSK: Corneal stromal keratocyte
DAPI: 4′,6-diamidino-2-phenylindole
DAVID: Database for annotation, visualization and integrated discovery
DMEM: Dulbecco’s modified Eagle medium
ECM: Extracellular matrix
ELISA: Enzyme-linked immunosorbent assay
ESI: Electrospray ionization
F-AME: Fresh amnion extract
FBS: Fetal bovine serum
FDR: False discovery rate
GO: Gene ontology
IDA: Information-dependent acquisition
IGF1: Insulin-like growth factor 1
iTRAQ: Isobaric tagging for relative and absolute quantitation
KEGG: Kyoto encyclopedia of genes and genomes
MS: Mass spectrometry
NGS: Normal goat serum
PBS: Phosphate buffered saline
r.p.m.: Round-per-minute
ROCK: Rho-associated coiled-coil containing protein kinase
SD: Standard deviation
SDS-PAGE: Sodium dodecylsulfate-polyacrylamide gel electrophoresis
TGF: Transforming growth factor
TIMP1: Tissue inhibitor of metalloproteinase 1
